# Electronic decay of core-excited HCl molecules probed by THz streaking

**DOI:** 10.1063/1.5091756

**Published:** 2019-05-08

**Authors:** K. Wenig, M. Wieland, A. Baumann, S. Walther, A. Dimitriou, M. J. Prandolini, O. Schepp, I. Bermúdez Macias, M. Sumfleth, N. Stojanovic, S. Düsterer, J. Rönsch-Schulenburg, E. Zapolnova, R. Pan, M. Drescher, U. Frühling

**Affiliations:** 1 Institute for Experimental Physics, University Hamburg, Hamburg, Germany; 2 The Hamburg Centre for Ultrafast Imaging - CUI, Hamburg, Germany; 3 Deutsches Elektronen-Synchrotron - DESY, Hamburg, Germany

## Abstract

The ultrafast electronic decay of HCl molecules in the time domain after resonant core excitation was measured. Here, a Cl-2p core electron was promoted to the antibonding σ^*^ orbital initiating molecular dissociation, and simultaneously, the electronic excitation relaxes via an Auger decay. For HCl, both processes compete on similar ultrashort femtosecond time scales. In order to measure the lifetime of the core hole excitation, we collinearly superimposed 40 fs soft x-ray pulses with intense terahertz (THz) radiation from the free-electron laser in Hamburg (FLASH). Electrons emitted from the molecules are accelerated (streaked) by the THz electric field where the resulting momentum change depends on the field's phase at the instant of ionization. Evaluation of a time-shift between the delay-dependent streaking spectra of photo- and Auger electrons yields a decay constant of (11 ± 2) fs for LMM Auger electrons. For further validation, the method was also applied to the MNN Auger decay of krypton. Reproduction of the value already published in the literature confirms that a temporal resolution much below the duration of the exciting x-ray pulses can be reached.

## I. INTRODUCTION

When an electron of a molecule is resonantly excited to an antibonding orbital, the molecule will start to dissociate, and if the excitation has created a core-hole, the latter will typically relax electronically. Electronic de-excitation via the emission of an Auger electron is usually very fast and occurs within a few femtoseconds, while in most cases, the nuclear rearrangement is much slower. Thus, both processes can often be studied separately. However, for molecules containing a very low mass component such as the hydrates HI, HBr, and HCl, the dissociation is also very fast and may evolve on similar time scales as the Auger-emission. This was first observed in HBr molecules after resonant excitation of 3d electrons to the antibonding 4σ^*^ orbital.^1^ The emitted Auger electrons were found to exhibit a narrow, atomiclike spectrum, thus indicating emission of the majority of the electrons after the dissociation. This energy-domain technique of deducing the dynamics of a process by relating it to the exponential decay of a core excitation is commonly known as the core hole clock method and has been used for decades to study molecular dissociation rates,^2^ charge transfer processes,^3^ and wave packet dynamics.^4^ The speed of the electronic decay, which sets the clock in these experiments, can be easily obtained for pure atomic Auger electrons where the excited state lifetime can be inferred from spectral widths. This is no longer possible for Auger electrons emitted from molecules where the spectra are broadened due to nuclear motion. For these systems, a time domain approach may remove such limitations.

The first time domain measurement of an Auger decay was performed in krypton atoms.^5^ The atoms were ionized by attosecond XUV pump-pulses from a high-harmonic source, and the energy of the emitted Auger electrons was modulated (streaked) by an ultrashort, few femtosecond near-infrared (NIR) laser probe-pulse. By scanning the delay of the pump- and probe-pulse, the Auger lifetime of $7.9_{-0.9}^{+1}$ fs was measured. Later, experiments measured Auger lifetimes by using charge state chronoscopy^6,7^ and recently by transient absorption spectroscopy.^8^ All these experiments rely on attosecond pump- and intense few femtoseconds NIR probe-pulses. The generation of attosecond pulses is experimentally very challenging and limited to the XUV regime, if decent photon flux is required. Another difficulty in these experiments is the high intensity of the NIR probe pulses. On the one hand, these NIR pulses can perturb the system under study^8^ and, on the other hand, can lead to a significant background signal due to multiphoton ionization.

Here, we present an alternative way to measure the Auger lifetime by using femtosecond pump-pulses and long-wavelength THz radiation to streak the emitted electrons. The system under study is excited by short soft-x-ray pulses, and the emitted electrons are accelerated by a superimposed THz-light field with an oscillation period much longer than the investigated processes. The interaction with the THz field causes a time-dependent momentum change *Δp* of the electrons according to

$$\Delta p\left(t_{i}\right)=-\int_{\mathrm{ti}}^{\infty }e\;E\left(t\right)dt=-e\;A\left(t_{i}\right)=-\mathrm{eA}(\varphi \left(t_{i}\right)),$$ (1)

where *A(t_i_*) is the vector potential at ionization time *t_i_*, $\varphi \left(t_{i}\right)\;\mathrm{is}\;$ the phase of the electric field, and *e* is the electron charge. When the relative delay between the electron emission time and the THz streak-field is scanned, the evolution of the streak field is mapped onto changes of electron energies. The electron emission time *t_i_* is encoded in the phase of the streak curve.

Photoelectrons are emitted instantaneously after absorption of the ionizing photons, and thus, the temporal profile of the photoelectron emission rate represents the temporal profile of the ionizing light pulse. The temporal profile of the Auger emission can be described as a convolution of the temporal distribution of the core hole creation and the evolution of the Auger process. Here, we assume a soft x-ray pulse with a Gaussian intensity envelope $I_{X}=e^{-\frac{t^{2}}{2\sigma_{X}^{2}}}$ and an exponential Auger decay

$$\mathrm{AE}\left(t\right)=\left\{\begin{array}{c} A_{1}e^{-(t-t_{0})/\tau_{\mathrm{AE}\;}},\\ 0, \end{array}\;\begin{array}{c} t\geq t_{0}\\ \;t<t_{0}, \end{array}\right.$$ (2)

where $\sigma_{X}\;$ corresponds to the root mean square (rms) pulse duration and $A_{1}$ corresponds to the initial number of core vacancies. The parameter $t_{0}$ accounts for a delayed starting time for the decay. The Auger emission rate is then given by

$$I_{\mathrm{Auger}}\left(t\right)=I_{X}(t)⊗\mathrm{AE}\left(t\right)=\frac{2\sqrt{\;2}A_{1\;}\sigma_{X}}{\sqrt{\pi }}\;\cdot e^{1/\tau_{\mathrm{AE}\;\cdot }\left(\frac{\sigma_{X}^{2}}{2\tau_{\mathrm{AE}}}-t-t_{0}\right)\;}\cdot \left[1-\mathrm{erf}\left(\frac{1}{\sqrt{2}\sigma_{X}}\left(t_{0}-t-\frac{\sigma_{X}^{2}}{\tau_{\mathrm{AE}}}\right)\right)\right].$$ (3)

**FIG. 1.**
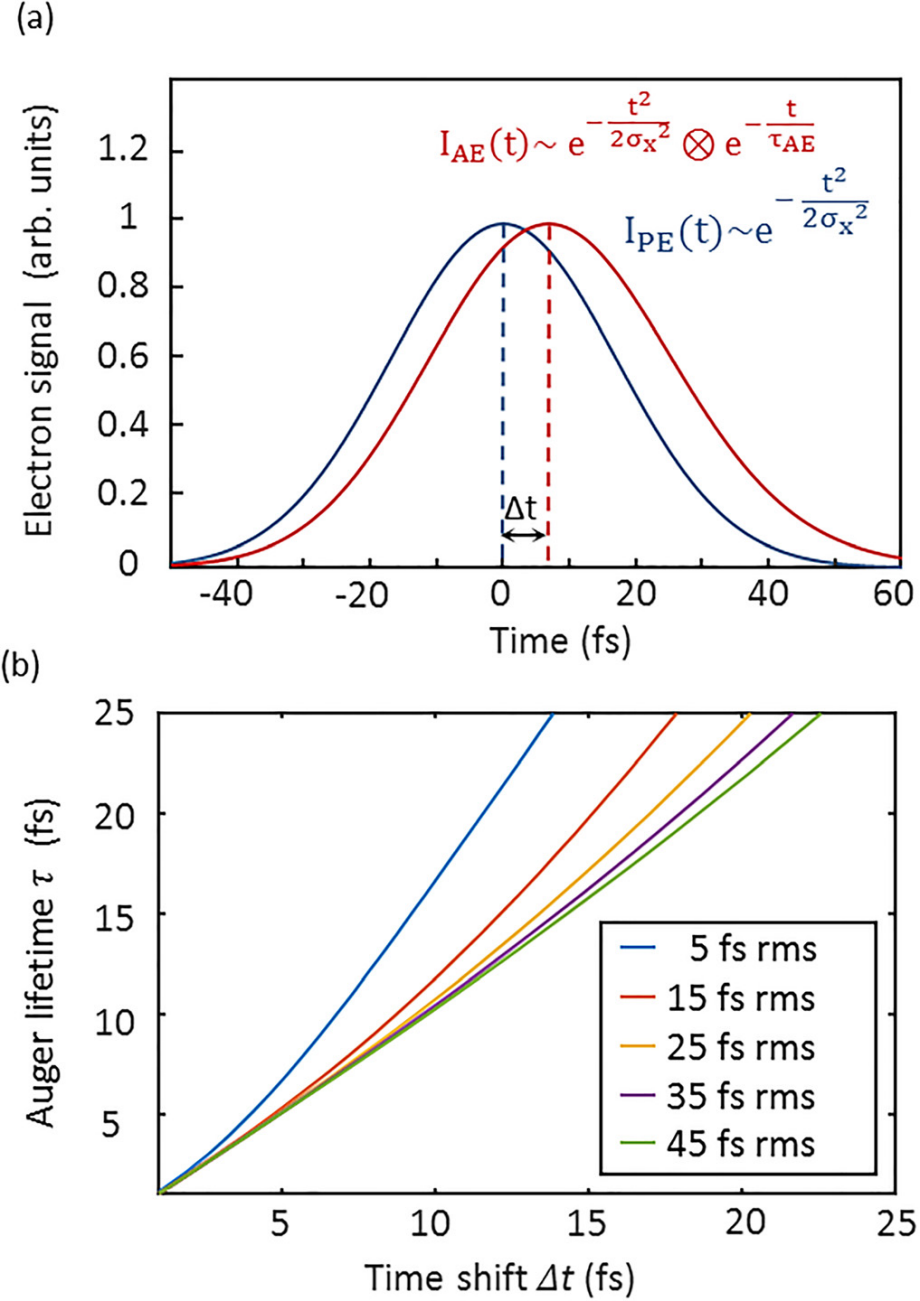
(a) Emission rates of photo- (black) and Auger electrons (red) generated by an x-ray pulse with a Gaussian envelope and a rms pulse duration of 17 fs (40 fs FWHM). As an example for krypton, the value of the Auger lifetime is here set to 7.9 fs with *t_0_* = 0. Due to the on average later emission of the Auger electrons, the peak of the Auger emission is shifted by *Δt* with respect to the maximum of the photoelectron emission. (b) The Auger lifetime τ_AE_ as a function of the time shift *Δt* of the Auger emission peak for different rms x-ray pulse durations. The shift was determined by fitting Gaussian curves to the Auger emission rates calculated using Eq. (3) with *t_0_* set to zero.

Figure 1(a) depicts the calculated photo- and Auger electron emission rates generated by a light pulse of 17 fs rms duration (40 fs FWHM) and an Auger lifetime of $\tau_{\mathrm{AE}}=7.9\;\mathrm{fs}.$ For x-ray pulse durations much longer than the Auger lifetime, the shape of the Auger emission rate is mainly determined by the shape of the ionizing light pulse. However, in spite of an instantaneous start of the decay ($t_{0}$ = 0), due to the finite lifetime $\tau_{\mathrm{AE}}$, the maximum of the Auger emission is delayed by *Δt* with respect to the maximum of the photoelectron emission. The shift depends on the Auger lifetime, the x-ray pulse duration, and the onset $t_{0}$ of the decay [see Fig. 1(b)].

Owing to this shift *Δt*, the on-average delayed Auger electrons are exposed to a different phase of the streak field than the photoelectrons. This is visible as a phase shift between the photo- and Auger electron streak spectrograms. Phase shifts much smaller than the duration of the ionizing pulses become discernible. Therefore, lifetimes much shorter than the duration of the exciting pulses can be measured. This technique is regularly used in attosecond physics, where, e.g., photoemission delays of (21 ± 5) as have been observed after ionization with 200 as XUV pulses and streaking with NIR.^9–11^ To obtain meaningful streak spectra, streak-fields with oscillation periods long compared to both the processes studied and the ionizing light pulses have to be used. NIR at an 800 nm wavelength has an oscillation period of only 2.5 fs and is suitable to study processes in the attosecond range. By choosing a streak-field in the far infrared (THz) region with an oscillation period of 177 fs, we have transferred this technique to the femtosecond range.^12^ We found a THz electric field of only 5 MV/m to be sufficient for the streaking, which is about three orders of magnitude below the field strengths commonly used in NIR streaking experiments.

First, to prove the ability and test of the accuracy of the experiment, the lifetime of the well-studied MNN Auger decay in krypton has been measured. Subsequently, the characteristic time constant of dissociating HCl molecules after resonant excitation of 2*p*_Cl_ electrons to the antibonding σ^*^ orbital was investigated, which to our knowledge is the first time-resolved measurement of this process.

## II. EXPERIMENT

The experiment has been performed at the free-electron laser in Hamburg FLASH.^13,14^ A detailed description of the setup can be found in Ref. 12. Briefly, the soft x-ray pulses provided by FLASH are collinearly superimposed with intense 53 *μ*m THz pulses from the FLASH THz undulator.^15^ Both pulses are inherently synchronized due to their generation from the same electron bunch. The 200 eV soft x-ray pulses were focused with a Cr/C multilayer mirror with a focal length of 2 m to a spot of about 100 *μ*m diameter. The peak reflectivity of the mirror was 15% at a wavelength of 6.19 nm (200.3 eV) with a FWHM bandwidth of 2.8 nm (2.4 eV). The spectrum of the soft x-ray pulses was measured with the FLASH VLS spectrometer.^16^ The width of the spectrum averaged over 5000 shots was 0.05 nm (1.5 eV) FWHM with a central wavelength within the mirror reflectivity range.

The THz pulses with an initial pulse energy of 1–2 *μ*J and a pulse duration of 1.8 ps were focused using an off-axis parabolic mirror with a focal length of 200 mm. To filter out harmonics and smooth the evolution of the THz field, a THz bandpass filter was used. The electric field-strength in the THz focus was 5 MV/m, which was determined from the streaking amplitude. The delay between the two pulses was adjusted by a delay stage with a step size of 1 *μ*m. The target gases were provided by a pulsed nozzle (Parker Series 9 solenoid valve). The target density was set carefully in order to avoid space-charge effects with chamber pressure being lower than 5 × 10^−6^ mbar. The electron spectra were recorded by two time-of-flight spectrometers (TOFs) aligned in the plane of the THz polarization. The polarization of the soft x-ray pulses was perpendicular to the polarization of the THz light.

The duration of the soft x-ray pulses was in the range from 30 to 50 fs (FWHM). For each run of measurement, the average x-ray pulse duration was determined by comparing the width of unperturbed electron spectra with those taken close to a zero crossing of the THz vector potential. Assuming a Gaussian envelope of the averaged x-ray pulses, the rms pulse duration can be calculated by^17^

$$\sigma_{X}=\sqrt{\frac{\sigma_{\mathrm{streak}}^{2}-\sigma_{0}^{2}}{s^{2}}},$$ (4)

where *σ_streak_* and $\sigma_{0}$ correspond to the rms widths of the streaked and the field-free electron energy spectrum, respectively, which were obtained by fitting with Gaussian curves. The streaking speed *s* is defined by the temporal derivative of the electron energy change $\Delta W_{\left|\right|}\;$ due to the streaking field

$$s=\frac{\partial \left(\Delta W_{∥}\right)}{\partial t}.$$ (5)

The streaking speed is assumed to be constant close to zero crossings of the vector potential and was determined by a linear fit to the centers-of-mass of the photo- and Auger electron peaks in the vicinity of the zero crossings. In our case, streaking speed values in the range of 0.01 to 0.04 eV/fs were measured. By comparing streak spectra taken by the two TOFs facing each other, a possible linear chirp of the soft x-ray carrier frequency can be disclosed. During the measurements, no significant chirp was observed.

## III. RESULTS

### A. Auger decay in krypton

**FIG. 2.**
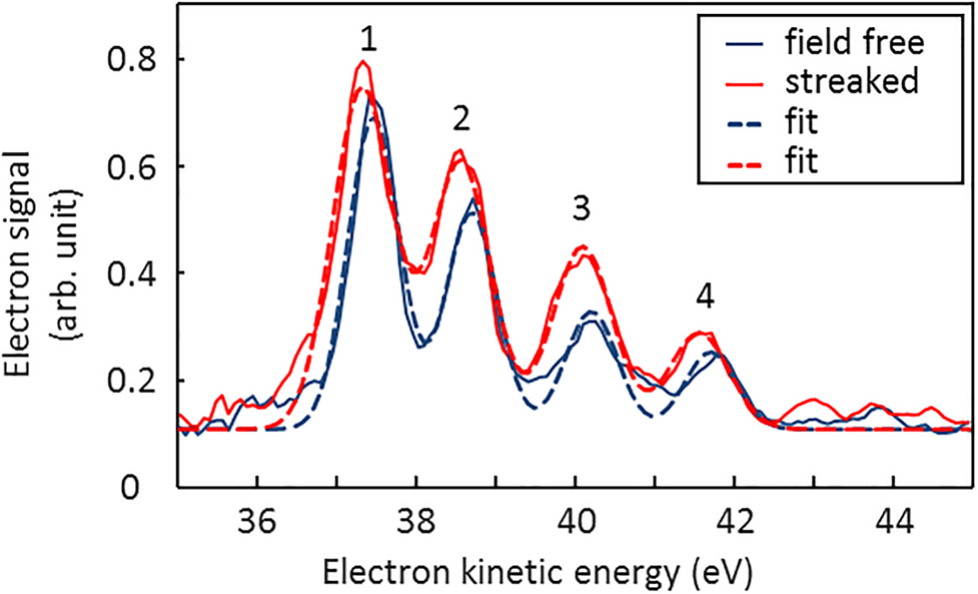
Krypton M_4,5_N_1_N_2,3_ Auger spectra measured without (blue) and with (red) the terahertz streak field with fitted Gaussian curves. The spectra were averaged over 100 shots. From the fits, the energy shift *ΔE* of the streaked electrons with respect to the field-free spectrum was obtained.

In order to prove the capability of our setup, we investigated the well-studied atomic Auger decay in krypton as a benchmark experiment. To this end, krypton atoms were ionized with 200 eV photons. With this photon energy, it is possible to ionize krypton 4p, 4s, and 3d electrons. Upon photoionization of 3d electrons, the generated core hole relaxes via emission of MNN Auger electrons. Figure 2 depicts averaged kinetic energy spectra of the M_4,5_N_1_N_2,3_ krypton Auger electrons in the range of 35 eV to 43 eV measured with (solid red line) and without the THz streak field (solid blue line). Here, the delay between the THz and x-ray pulses was set close to a zero crossing of the THz streak field.

The dashed lines represent Gaussian curves fitted to the Auger peaks. Here, as a boundary condition for the fit, the rms widths of the M_5_N_1_N_2,3_ (first) and M_4_N_1_N_2,3_ (second and fourth) peaks were set to be equal. The third peak contains two lines with an energy difference of 0.1 eV^18^ which cannot be resolved. Thus, the position and width of this line were chosen to be free parameters. Also, a constant background was included as a fit parameter. From the streak broadening of the Auger M_5_N_1_N_2,3 _line, taken from the fits, a mean duration of the electron distribution of (44 ± 5) fs FWHM was determined. As discussed above, the temporal profile of the Auger electron emission consists of a convolution of the temporal profile of the ionizing x-ray pulse and an exponential function describing the Auger decay (see Fig. 1). A deconvolution of the well-known 7.9 fs Auger lifetime yields an x-ray pulse duration of (41 ± 5) fs.

**FIG. 3.**
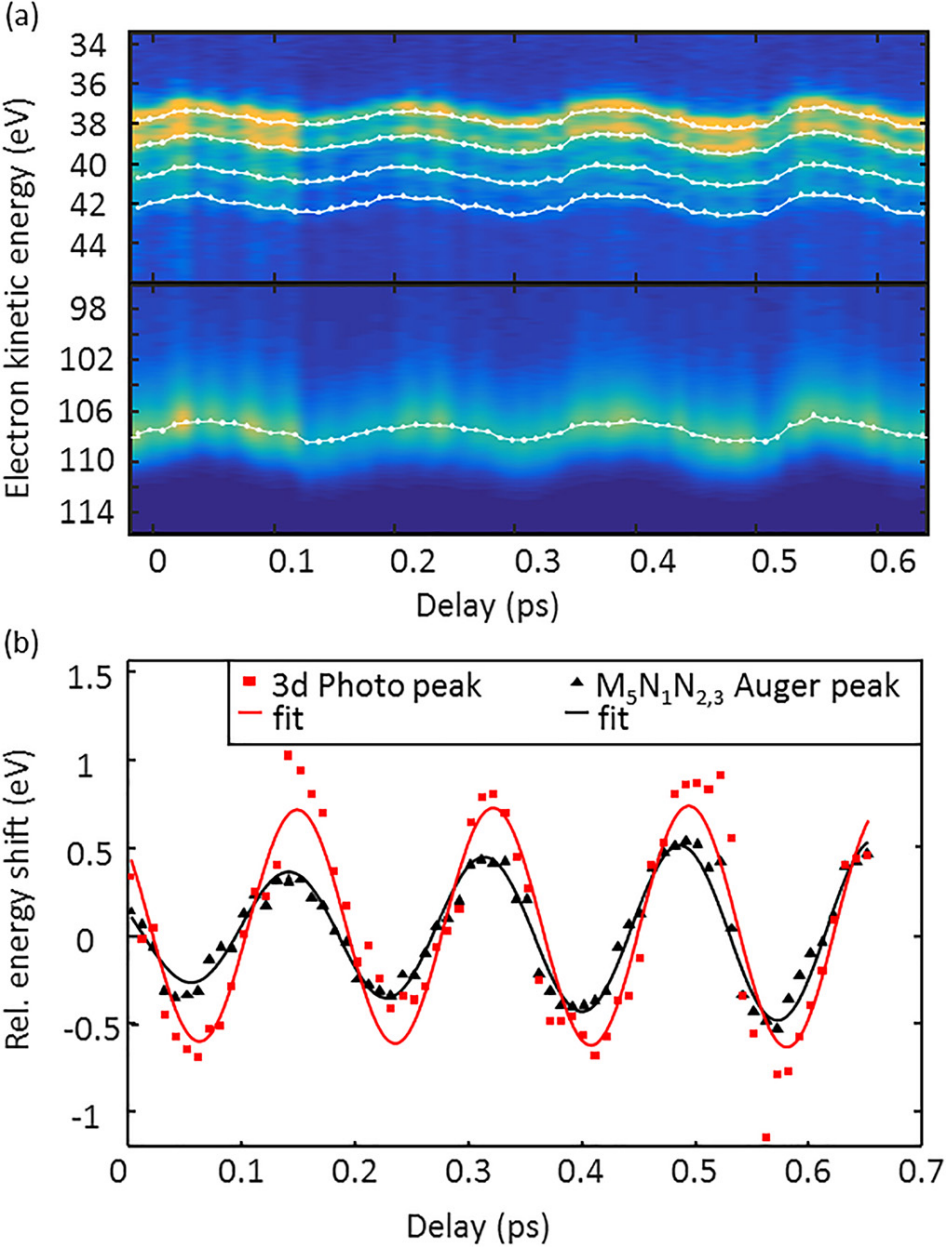
(a) Series of krypton Auger (upper panel) and photoelectron (lower panel) spectra simultaneously taken for different THz/x-ray delay times. Each spectrum was averaged over 100 shots, and the overall intensity was normalized. The white dots represent the maxima of Gaussian curves fitted to the spectra. The 1.2 eV spin–orbit splitting of the 3p_5/2_ and 3p_3/2_ photoelectrons is not resolved by the TOF-spectrometers. (b) Sinusoidal curves (solid lines) are fitted to the energy shift of the photo- and Auger electron lines of the spectra. The phase shift between the Auger and photoelectron curves reflects the averaged delayed emission of the Auger electrons. For this particular scanning trace, a delay of (7 ± 9) fs was determined from the sinusoidal fits.

Figure 3(a) displays a series of streaked electron spectra for different THz/x-ray delay times. The oscillation of the THz vector potential is mapped onto changes of the recorded electron energies. As indicated in Fig. 1, the maximum of the Auger temporal profile is shifted with respect to the maximum of the photoelectrons by *Δt*. This temporal delay is visible in the streak scan as a phase difference between the streak-oscillations of the direct photo- and the Auger electrons. To precisely determine this phase difference, Gaussian curves were fitted to the photo- and Auger electron lines of each spectrum, and their energy shift with respect to the field-free spectrum was calculated (see Fig. 2). Plotted in Fig. 3(b) are the thus obtained energetic shifts due to streaking. Owing to a higher initial kinetic energy of the 3d photoelectrons (W_0,PE_ = 107.5 eV) compared to the Auger electrons (W_0,AE_ = 37.7 eV), the streak amplitude is a factor of $\sqrt{W_{0,\mathrm{PE}}/W_{0,\mathrm{AE}}}=$ 1.7 higher for the photoelectrons. By fitting sinusoidal curves to the data, a time difference between the two functions of *Δt* = (7 ± 5) fs was determined as the weighted average of two scanning trace measurements, where both electron spectrometers were evaluated.

In an alternative evaluation procedure, electron spectra measured with both TOFs at three different time delays close to zero crossings of the THz vector potential were analyzed. The shift of the photo- and Auger electron spectra with respect to the field-free spectrum was evaluated for each measurement trace. Here again, the peaks were fitted by Gaussian functions. At the zero crossing, we assume the energy change to be linear in time and calculate the mean ionization time $t_{i}=\Delta E/s$ for each peak, where *ΔE* is the energy shift of the investigated spectral line and *s* is the corresponding streaking speed [see Eq. (5)]. For this evaluation procedure, a time difference of *Δt* = (6 ± 5) fs between the maxima of the M_5_N_1_N_2,3_ Auger and the 3d photoelectron peak as the weighted average over all measurements at the three different zero crossings was obtained.

Using Eq. (3), the estimated pulse duration, and the weighted average of the determined time shifts, the values corresponding to an Auger lifetime of *τ* = (8 ± 5) fs are in agreement with the literature.^5^ The error of the Auger lifetime is dominated by the error in the determination of the shift *Δt* and could be reduced by improving the measurement statistics.

### B. Auger decay in HCl

Owing to the hydrogen's low mass, the dissociation of HCl after resonant excitation of 2p_Cl_ electrons to the antibonding $\sigma^{*}$ orbital is fast compared to the electronic relaxation via Auger decay. Thus, the majority of the Auger electrons are emitted from chlorine atoms rather than HCl molecules. This is reflected in sharp atomiclike Auger lines.^19,20^ However, the Auger spectra are not purely atomic because some electrons are also emitted before and during the dissociation. The kinetic energy distribution of these electrons is broad and shifted to smaller energies with respect to the atomic Auger lines.^21,22^ The precise shape of the spectrum depends on the dissociation speed and thus on the energy of the exciting photons.^23^ While the energy of the emitted Auger electrons is clearly influenced by the nuclear dynamics, we assume the lifetime of the core hole excitation to be independent of the internuclear distance of the Cl and H atoms and to be in good approximation equal to the lifetime of the 2p_3/2_ hole in the Cl^*^ atom.^21,24^

**FIG. 4.**
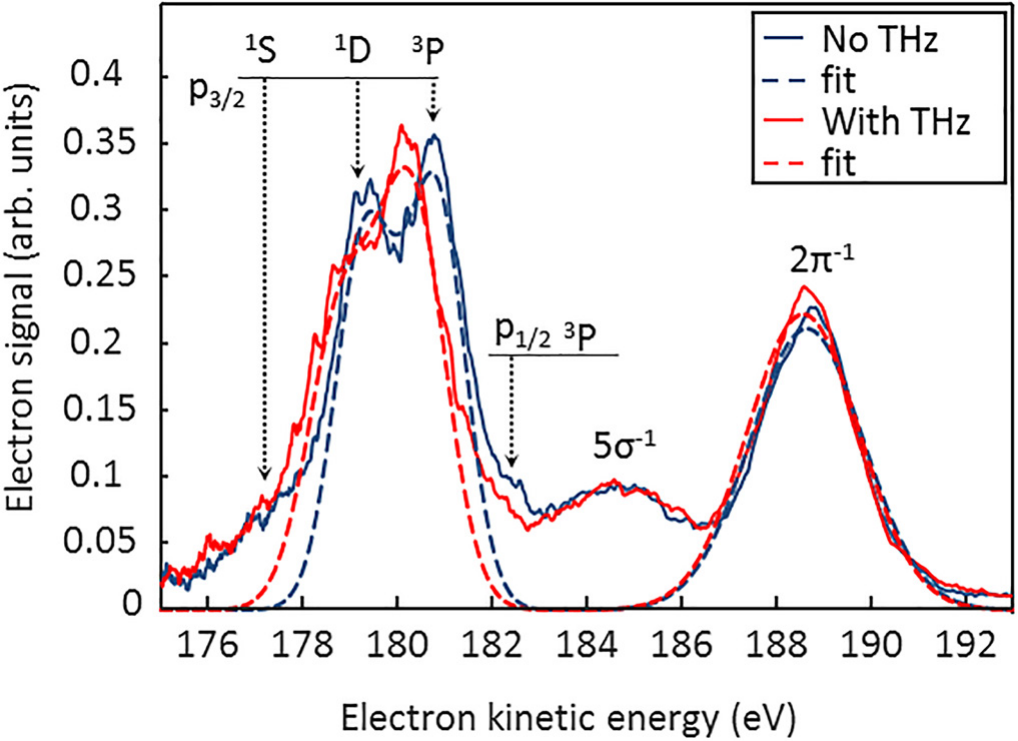
Photo- and Auger electron spectra from HCl molecules measured without (blue) and with (red) the terahertz-streak field. The dashed lines indicate Gaussian curves fitted to the investigated peaks in order to obtain the energy shift *ΔE* of the streaked electrons with respect to the field free case. Each spectrum is averaged over 600 shots.

Figure 4 depicts streaked (red) and THz-field-free (blue) electron spectra of HCl molecules measured after resonant excitation with (200.0 ± 1.5) eV photons. These spectra are each integrated over 600 single shots. In the non-streaked spectra, the lines at 179 eV and 180.5 eV correspond to the atomic chlorine Auger lines from the $2p_{3/2}^{5}$ 3p^6^ → 3p^4^ (^1^D) and $2p_{3/2}^{5}$ 3p^6^ → 3p^4^ (^3^P) transitions and are clearly resolved, whereas in the streaked spectrum, these Auger lines overlap due to the broadening induced by the THz field. The feature at 182 eV is attributed to 2p_1/2_ (^3^P) Auger electrons. Due to the broad excitation photon energy of 1.5 eV, this line contributes as well to the average spectrum. The Auger decay from the $2p_{3/2}^{5}$ 3p^6^ → 3p^4^ (^1^S) at 177 eV is not resolved. Here, the atomic Auger peak overlaps with contributions of molecular Auger electrons. The peaks at 184 eV and 188 eV belong to the photoelectrons of the 5σ^−1^ and 2π^−1^ orbitals. The dashed lines mark Gaussian curves which are fitted to the investigated peaks. As a restriction to the fit parameters, the widths of the atomic Auger peaks are assumed to be equal, and their distance is fixed to *dE* = 1.5 eV taken from Ref. 20. We estimated the total amount of the molecular Auger electrons by calculating the difference of the measured spectra and the fitted Gaussian curves to (26 ± 15) %. From the broadening of the 2π^−1^ photoelectron peak, a pulse duration of (40 ± 5) fs *(FWHM)* was determined which confirms the pulse duration obtained from the broadening of the krypton Auger line.

**FIG. 5.**
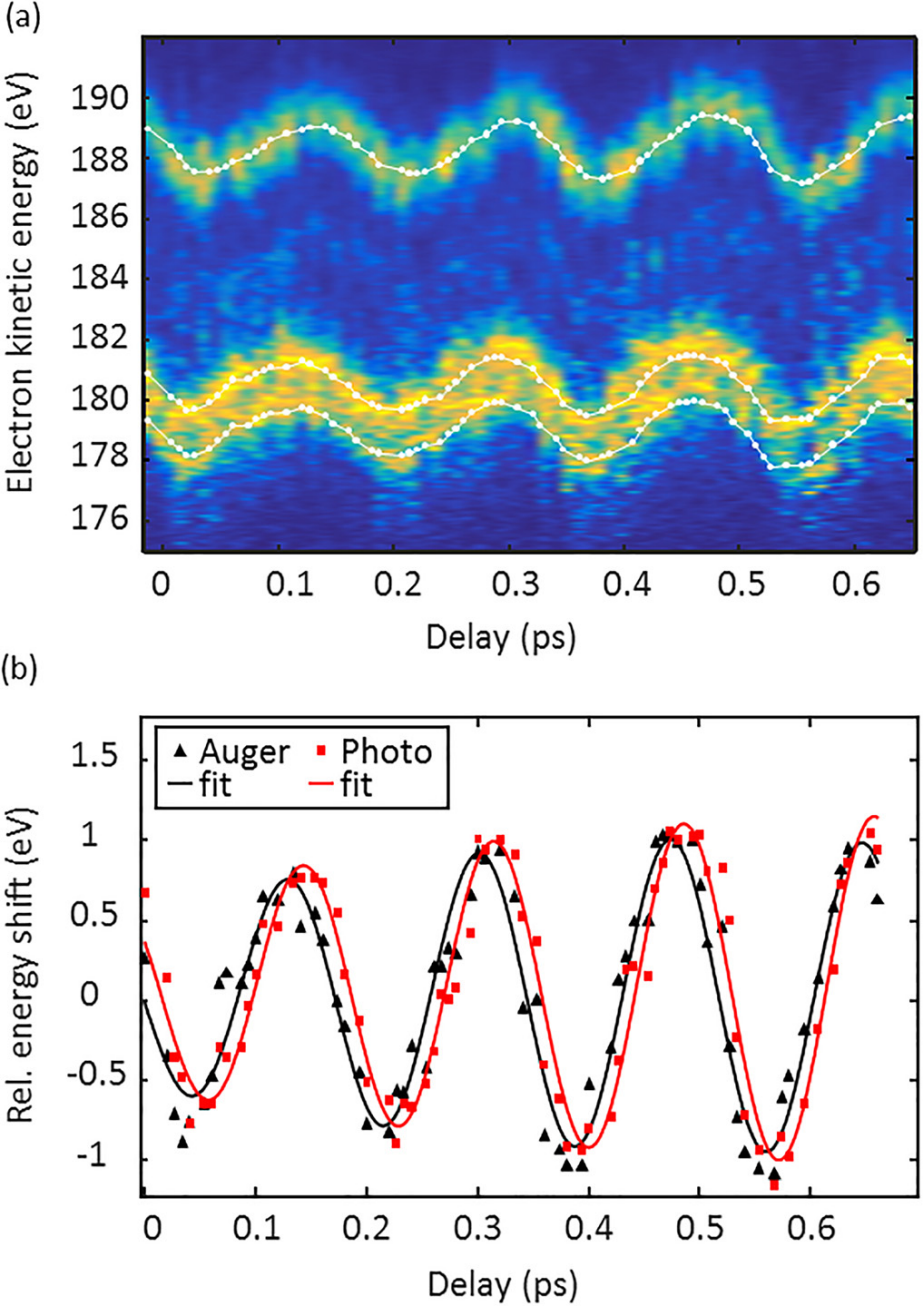
(a) Series of HCl photo- and Auger electron spectra taken for different THz/x-ray delay times. The white dots mark the center of the 2π^−1^ photoelectron (upper line), and the ${2p}_{3/2}$ ^1^D and ${2p}_{3/2}$ ^3^P Auger electron peaks (lower lines) are taken from the fitted Gaussian curves. Each spectrum is an averaged over 100 shots and is intensity-normalized. (b) Sinusoidal curves (solid lines) are fitted to the energy shift of the photo- and Auger electron lines of the spectra. The visible phase shift between them reflects the on- average later emission of the Auger electrons with respect to the photoelectrons. For this particular trace, the determined delay is (15 ± 7) fs.

Figure 5 shows a series of HCl photo- and Auger electron spectra taken for different THz/x-ray delay times. At each delay position, 100 shots are averaged. The phase-dependent energy-modulation of the ^1^D and ^3^P Auger electron and the 2π^−1^ photoelectron lines is clearly visible. The peaks are fitted with Gaussian functions, and the white dots mark the resulting peak centers. For a quantitative analysis, a sinusoidal function is fitted to the center of the 2p_3/2_ (^1^D) Auger and the 2π^−1^ photo line. A time shift of Δt = (12 ± 3) fs is discernible from the difference of the respective fitting parameters. This is the weighted mean of eight scanning traces where both electron spectrometers were evaluated. Again, we additionally determined the time shift by evaluating the energy shift of streaked Auger and photoelectron peaks with respect to the field-free peaks measured close to a zero transition of the THz vector potential. Here, a time shift of Δt = (15 ± 4) fs was obtained. The value is a weighted average of data taken at ten different zero-crossing points with TOF spectrometers. The measurement error is dominated by the uncertainty of the streaking speed *s*. For further calculation, the weighted average of both methods is used.

The time shift given here was evaluated for the atomic Auger lines. As estimated above, (26 ± 15) % of the Auger electrons are emitted in the molecular regime. To obtain the lifetime of the Cl^*^ core hole excitation, we follow the model of Menzel *et al.*^21^ and assume an exponential Auger decay rate with a lifetime τ_Cl_ independent of the nuclear motion. The energy of the Auger electrons depends on the internuclear distance R at the emission time. The first electrons are emitted while R is small and are energetically shifted with respect to the Auger electrons emitted at later times with large R values. Thus, the emission of Auger electrons with energies corresponding to the atomic lines sets in with a delay t_0,atomic_, which can be understood as the dissociation time of the molecules.

Assuming a pure exponential decay as in Eq. (2), we can calculate $t_{0,\mathrm{atomic}}=-\ln\left(1-0.26\right)\tau =0.3\;\tau$. Inserting this as $t_{0}$ in Eq. (3), we deduce the corresponding Auger lifetime $\tau_{\mathrm{Cl}}=(11\pm 2)\;\mathrm{fs}$ for the determined time shift and pulse duration. The error of the estimated decay time starting point $t_{0}$ is rather large (±60%); however, the obtained value for *τ* only slightly depends on $t_{0}$. Note that including t_0,atomic_ in Eqs. (2) and (3) is an approximation which is applicable in the case discussed here, where the main contribution to the error of *τ* is given by the uncertainty in the determination of the time shift *Δt*.

## IV. CONCLUSION AND OUTLOOK

We have introduced the measurement of time-shifts in terahertz streak spectrograms to study ultrafast electronic decays and applied it to investigate the Auger decay in krypton atoms and HCl molecules. The measured lifetime for the krypton MNN Auger decay of *τ* = (8 ± 5) fs is in very good agreement with previous studies.^5^ For HCl molecules, the lifetime broadening of the 3s3p^5^ Auger lines was measured after resonant excitation with 200.85 eV photons to be (96 ± 5) meV, which corresponds to a lifetime of 7 fs.^25^ In contrast to the 3s^2^p^4^ Auger lines investigated here, these lines are almost purely atomic and thus facilitate such measurements. The value obtained by terahertz streaking of τ=(11±2) fs is significantly longer and also longer than the 2p_3/2_ core hole lifetime of isoelectronic argon atoms, which was inferred from the Auger linewidth to be only 5.9 fs.^26^ Though, it is in good agreement with the value of 12 fs estimated by Menzel *et al.* using a quasiclassical model.^21^

The lifetime presented in this work is the combined emission time for the ^1^D and ^3^P Auger electrons. Within our experimental resolution, no difference for the two lines was discernible. The measurement error is dominated by the uncertainty in the determination of the time shift between streaked photo- and Auger electron peaks. It was large in the case of krypton due to low statistics since only two streak traces with a moderate signal to noise ratio were measured in the available time window of approximately 2 h. If the x-ray pulse duration is long compared to the Auger lifetime, as is the case here, a variation of the pulse duration has only a small impact on the determined time constant. For example, in the case of krypton with τ = (8 ± 5) fs and an x-ray pulse of 40 fs FWHM, a change of 50% of the pulse duration results in a change of only 3.4% of the value determined for the Auger lifetime.

The presented approach based on the evaluation of phase shifts between photo- and Auger-electrons in streaking traces facilitates time resolved measurements with a resolution much better than the duration of the exciting light pulses. This makes it well suited for experiments at free-electron lasers where the pulse durations are often in the range of several tens of femtoseconds.^13,27^ Until now, time resolved investigations of Auger electron emission have mainly employed laser-based high-harmonic generation sources delivering ultrashort light pulses in the VUV to XUV range at photon energies usually below 100 eV. Using FEL sources will extend the usable spectrum to the x-ray regime and thus permit access to deep core-hole excitations. Since spectral shifts are evaluated rather than the width or shape of the Auger electron spectra, the technique is also applicable to the measurement of molecular Auger decay, where the spectrum is broadened due to nuclear motion.
